# The blending effect of natural polysaccharides with nano-zirconia towards the removal of fluoride and arsenate from water

**DOI:** 10.1098/rsos.221514

**Published:** 2023-03-08

**Authors:** M. Shanika Fernando, A. K. D. V. K. Wimalasiri, Karolina Dziemidowicz, Gareth R Williams, K. Rasika Koswattage, D. P. Dissanayake, K. M. Nalin De Silva, Rohini M. De Silva

**Affiliations:** ^1^ Centre for Advanced Materials and Devices (CAMD), Department of Chemistry, University of Colombo, Colombo 00300, Sri Lanka; ^2^ UCL School of Pharmacy, University College London, 29–39 Brunswick Square, London WCIN 1AX, UK; ^3^ Faculty of Technology, University of Sabaragamuwa, 70140 Belihuloya, Sri Lanka

**Keywords:** zirconia, chitosan, adsorption, fluoride, arsenate

## Abstract

Nano-zirconia (ZO) was synthesized using a microwave-assisted one-pot precipitation route. Two biopolymers, chitosan (CTS) and carboxymethyl cellulose were blended with ZO at different w/w ratios. The formulation with 30% w/w chitosan (ZO-CTS) was found to give enhanced uptake of F^−^ and As(V). ZO and the most effective ZO-CTS system were characterized using Fourier transform infrared spectroscopy, scanning electron microscopy, X-ray diffraction and X-ray photoelectron spectroscopy. These confirmed the formation of a composite system containing nanoparticles of 50 nm in size, in which ZO was present in the amorphous form. It was observed that the combination of ZO with CTS improved the F^−^ and As(V) adsorption capacity most notably at pH 5.5. Fluoride adsorption by ZO-CTS followed the Freundlich isotherm model, with an adsorption capacity of 120 mg g^−1^. Adsorption of As(V) by ZO-CTS could be fitted with both the Langmuir and Freundlich isotherm models and was found to have a capacity of 14.8 mg g^−1^. Gravity filtration studies conducted for groundwater levels indicated the effectiveness of ZO-CTS in adsorbing As(V) and F^−^ at a pH of 5.5. The ability of the ZO-CTS in removing Cd(II) and Pb(II) was also investigated, and no such enhancement was observed, and found the neat ZO was the most potent sorbent here.

## 1. Introduction

Clean drinking water to everyone by the year 2030 is one of the major sustainable development goals of the United Nations. Contamination of water bodies by inorganic anions such as fluoride (F^−^), arsenate (As(V)) and heavy metal ions such as Pb(II) and Cd(II) has been frequently reported [1,2]. Some of the aforementioned contaminants are suspected to be possible causative factors for chronic kidney disease of unknown aetiology (CKDu) in Sri Lanka [3–8]. This work was conducted with the objective of finding a method to remove all of these ions simultaneously from water. Most of the existing water purification methods cannot be used cost-effectively to remove persisting non-biodegradable contaminants from water, particularly at the domestic scale. The process of adsorption has been identified as one of the most cost-effective methods for removing pollutants and many materials, in the forms of both bulk [9] and nanoscale particles [10,11], have been identified as effective adsorbents for Pb(II), Cd(II), As(V) and F^−^. However, the application of many of these adsorbents is not practical due to their slow adsorption characteristics and/or high cost. Thus, there is a huge demand for the development of non-toxic rapid sorbents. Here, we focused our attention on the development of nano-zirconia (ZO) incorporated into biopolymer composites derived from natural polysaccharides. Zirconium dioxide (zirconia) is an inert, non-toxic material with inherent chelating properties and high chemical stability [12–16]. It has also been reported to be an effective adsorbent for contaminants such as heavy metal ions [17–20], fluorides [21] and arsenate [17,22,23]. Synthesis of zirconium dioxide nanoparticles has been attempted by many researchers using approaches such as forced hydrolysis, hydrothermal methods [22], co-precipitation [14], sol–gel methods [24] and thermal treatment methods with capping agents [25]. Microwave-assisted synthesis [26] has been identified as perhaps the most beneficial, because it can produce small particles having high purity and a narrow size distribution within a short reaction time. Therefore, we used a microwave-assisted method to synthesize composites here. As biopolymers, chitosan (CTS) and carboxymethyl cellulose (CMC) were selected due to their chelating and biodegradable properties. Furthermore, these polymers are abundant and reported to be efficient sorbents [9,11,27–37]. We report the synthesis, optimization and sorbent characteristics of a series of nano-ZO-biopolymer composites.

## 2. Materials and methods

CTS (medium molecular weight, 85% deacetylated; Sigma Aldrich), CMC (low viscosity; Sigma Aldrich), zirconium nitrate (Merck), ammonium hydroxide solution, 25% (Sigma Aldrich), cadmium nitrate, 98% (Sigma Aldrich), lead nitrate, 99.5% (Merck) sodium fluoride, 99.5% (Merk) and sodium arsenate, 99% (Merck) were used in this study. All the solutions in fluoride adsorption studies were prepared and stored in polypropylene containers. All the chemicals used were analytical grade and used without further purification.

### 2.1. Synthesis of zirconia nanoparticles

ZO nanoparticles were synthesized using a modified microwave-assisted coprecipitation method [14,26] without using high temperatures or capping agents. In this method, a 5.0 M ammonia solution was added dropwise to 200 ml of aqueous 0.1 M Zr(NO_3_)_4_, at a rate of one drop per second. The pH was maintained at 9–10. Next, the solution was subjected to a microwave treatment (600 W) over a total exposure duration of 6 min. The microwave treatment was conducted in 20 s exposures with 10 s gaps after each irradiation. Following the microwave treatment, the precipitate was allowed to settle, separated and washed until it became pH neutral.

### 2.2. Synthesis of nanocomposites

For the synthesis of nano-ZO-embedded composites, an *in situ* precipitation method was used [28,38]. Optimization experiments were first performed, and initially the synthesis of ZO-CTS and ZO-CMC nanocomposites was attempted using different polymer/ZO mass ratios ranging from 4 to 100% w/w. The required quantity of CTS or CMC was dispersed in 200 ml of 0.1 M aqueous zirconium nitrate under vigorous stirring at 300 r.p.m., to make a homogeneous solution. A solution of 5.0 M ammonia (200 ml) was added dropwise to the mixture and the final solution subjected to microwave treatment as described above.

#### 2.2.1. Effect of composition on adsorption properties

Adsorption studies were conducted separately for Pb(II) and Cd(II), using 300 ppm solutions. For As(V) and F, 50 ppm solutions were used. The amount adsorbed was calculated by measuring the concentration remaining in solution after 30 min.

### 2.3. Characterization of the materials

Scanning electron microscopy (SEM) images were obtained using a Hitachi SU6600 microscope to investigate the surface morphology. Fourier transform infrared spectroscopy (FT-IR) was undertaken on AVATAR-320 instrument (Thermo Nicolet) over the wavenumber range between 500 and 4000 cm^−1^. The samples were mixed with KBr (sample: KBr = 1 : 10 w/w) and pressed into pellets for analysis. X-ray diffraction (XRD) was performed on a Bruker D8 Focus diffractometer using CuK*α* radiation over a 2*θ* range of 3–60°, with a step size of 0.02° and a step time of 1 s. X-ray photoelectron spectroscopy (XPS) analysis of the samples was performed before and after the adsorption using a K-alpha spectrometer (ThermoFisher Scientific UK) equipped with Al-K*α* (1486.6 eV) X-ray source. Samples were analysed in constant analyser energy mode with pass energy of 50–200 eV and step of 0.1 eV. In this experiment, the base pressure in the UHV chamber was below 2 × 10^−8^ Pa and the X-ray power was kept at 100 W to minimize radiation damage.

### 2.4. Batch adsorption studies

Experiments to investigate the adsorption of fluoride, arsenate, cadmium and lead, on ZO and Zr-CTS were carried out in polypropylene containers. All experiments were conducted at room temperature (27 ± 1°C) with continuous horizontal shaking at a rate of 200 r.p.m. and both the effect of time and the pH were investigated separately as explained below. In the isotherm studies, the solutions were agitated until they reached equilibrium. The solutions were then filtered and the residual concentrations of the ions analysed, with dilutions where necessary. All adsorption studies were performed in triplicate, and the mean values are reported. The equilibrium adsorption capacity was calculated according to the following equation:

$$\mathrm{qe}=\;\left(\frac{\mathrm{Co}-\mathrm{Ce}}{m}\right)\;V\;,$$

where qe is the equilibrium amount of anions adsorbed per unit mass of the adsorbent (mg g^−1^), Co and Ce are the initial and final anion concentrations, respectively, *m* is the mass of the adsorbent, and V is the volume of solution used for isotherm experiments. The correlation coefficient (*R*^2^) and square sum of error (SSE) (the square of the difference between experimental adsorption capacity and calculated adsorption capacity divided by the corresponding calculated adsorption capacity) were used to identify the best-fit isotherm models.

### 2.5. Adsorption studies for fluoride

A stock solution of fluoride (1000 ppm) was prepared by dissolving the required amount of NaF in deionized water. It was then diluted to the desired concentration. Tests were conducted to investigate the pH dependence of fluoride adsorption by varying the initial pH from 2 to 9. Test solutions containing 350 ppm fluoride (20.0 ml) with 0.04 g were used and the pH of the solutions was adjusted using aqueous 0.1 M HCl and 0.1 M NaOH. The effect of time on the adsorption capacity for fluoride was also investigated, using 20.0 ml of a 350 ppm fluoride solution at the optimum pH of 5.2 ± 0.1.

Batch adsorption studies were conducted at pH 5.2 in the concentration range from 40 ppm to 350 ppm using a fixed volume of 20.0 ml and an adsorbent dosage of 0.020 g. Residual fluoride concentrations were measured using a fluoride ion selective electrode (Hanna Instruments).

### 2.6. Adsorption studies for arsenate

A stock solution of arsenate (100 ppm) was prepared by dissolving the required amount of Na_2_HAsO_4_ in deionized water. It was then diluted to the desired concentration. The effect of pH for arsenic adsorption was investigated in between pH 2 and 9 using a 50 ppm of initial As(V) solution. The optimum contact time for As(V) adsorption was determined using 20.0 ml aliquots of arsenate solutions having 50 ppm concentration, pH of 5.5 and 0.04 g of adsorbent. Batch adsorption studies for arsenate were carried out using the same conditions in between 4 and 50 ppm of As(V) concentration range. The residual arsenic concentrations were measured using a GBC 932 AB graphite furnace atomic adsorption spectrometer (AAS).

### 2.7. Adsorption studies for Pb(II) and Cd(II)

Stock solutions (2000 ppm) of Pb(II) and Cd(II) were prepared in deionized water using lead nitrate and cadmium nitrate, respectively, and diluted as required. In order to investigate the equilibrium time, 0.04 g of adsorbent was added into 10.0 ml of 2000 ppm Pb(II) and 500 ppm Cd(II) solutions. The effect of pH was also investigated in a pH range of 2–9. Adsorption isotherm studies for Pb(II) were carried out within the concentration range from 300 to 2000 ppm while those for cadmium were undertaken from 100 to 600 ppm. All the experiments were conducted at the solution pH of 6.5 ± 0.1, without adding any acid or base. The concentrations of Pb(II) and Cd(II) were measured using a GBC 932 AB AAS with necessary dilutions. Low-level (ppb) ion concentrations were analysed using an Agilent 4219 microwave plasma atomic emission spectrophotometer.

### 2.8. Analysis of nano zirconia chitosan, nano zirconia and their fluoride and arsenate adsorbed samples by energy-dispersive X-ray spectroscopy

Energy-dispersive X-ray spectroscopy (EDEX) of ZO-CTS and ZO before and after adsorption of F^−^ and As(V) were also performed using a SEM-EDAX (Zeiss Gemini SEM 300) analyser, in order to identify the elemental compositions of ZO-CTS, ZO and F^−^, and As(V) adsorbed ZO-CTS and ZO (ZO-CTS-F, ZO-F, ZO-CTS-As and ZO-As).

### 2.9. Adsorption isotherms

Data obtained from the batch adsorption studies were fitted to the Langmuir, Freundlich, Temkin and Dubinin–Radushkevich (DR) adsorption isotherm models, as explained in previous work [39–43].

### 2.10. The effect of other ions

The effect of some of the most common coexisting anions like chloride, nitrate, nitrite, sulphate, phosphate, bicarbonate and hydroxyl ions on the adsorption of F^−^ and As(V) onto ZO-CTS were examined using 5 ppm and 50 ppb of initial concentrations, respectively, as these concentrations are more or less equal to the highest reported levels in groundwater in Sri Lanka [6,44]. The concentrations of the other ions in each solution were adjusted to two different concentrations (5 and 50 ppm) and adsorption studies were conducted using 20.0 ml of solution and 0.04 g of ZO-CTS separately for each solution.

### 2.11. Gravity filtration studies

Gravity filtration studies were conducted using groundwater samples spiked with F^−^ ions and As(V) ions to match their concentrations to the reported existing levels in the groundwater. The initial concentration of F^−^ and As(V) were adjusted to 3.6 ppm and 34 ppb as these concentrations are within the existing levels in groundwater in Sri Lanka. The study was conducted at two different pH levels, pH was 6.8 (the pH of the freshly prepared solution) and pH 5.5 (achieved by adding dilute HCl), the optimum pH for adsorption.

In order to investigate the applicability of ZO-CTS in adsorbing F^−^ and As(V) in gravity filtration, studies were conducted using a column with 1 cm diameter. Two hundred milligrams of ZO-CTS was loaded over a 50 mg cotton plug, and the multi-ion solution was passed through the column at a flow rate of 20 ml min^−1^.

### 2.12. Reusability test

For these experiments, the required solid phase was separated by filtration and the adsorbed ions were removed by washing with acid or base (20 ml for 0.01 g). Fluoride- and arsenate-adsorbed materials were magnetically stirred with 0.1 M NaOH at 300 r.p.m. for 3 h. The adsorbents were then washed well with deionized water until the pH of the washings become neutral. The regenerated materials were used for the adsorption studies using initial concentration of 3.6 ppm F^−^ and 34 ppb of As(V) concentrations with 0.04 g of dosage and 20.0 ml of volume. This process was repeated over five cycles

## 3. Results and discussion

### 3.1. Effect of the ratio of ZO : polymer on adsorption properties

**Figure 1.**
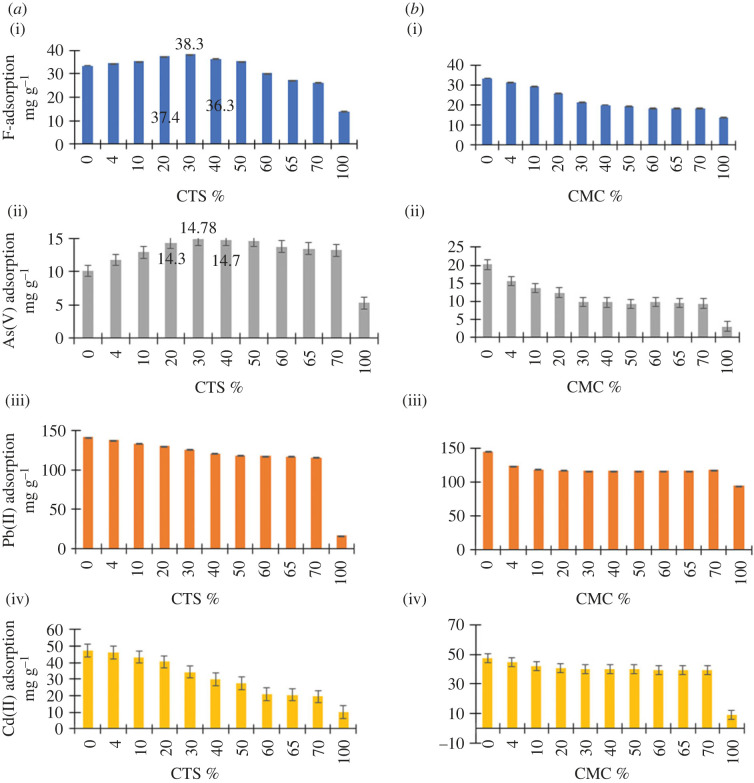
Effect of the % w/w polymer on the adsorption of F^−^, As(V), Pb(II) and Cd(II) by (*a*) ZO-CTS and (*b*) ZO-CMC nanocomposites. (i) F^−^, (ii) As(V), (iii) Pb(II) and (iv) Cd(II) uptake.

In order to identify the effect of the ZO : polymer ratio on the adsorption properties, a series of ZO-CTS and ZO-CMC formulations with varied w/w ratios were subjected to adsorption studies. Initial concentrations and dosages were selected in such a way that equilibrium is reached before complete adsorption of the selected ion. The results were plotted as the adsorption capacity versus percentage of CTS and CMC in the composite (figure 1*a*(i–iv) and *b*(i–iv)). The results indicated that the combination of CTS with nano-ZO improves the adsorption capacity for fluoride and As(V)). The pure polymers showed the lowest adsorption capacity for all the ions, with the composite containing 70% (w/w) ZO and 30% (w/w) CTS having the highest absorption capacity for fluoride and arsenate. However, the adsorption capacity of ZO-CTS for Pb(II) and Cd(II) is lower than that of pure ZO.

It can be observed that the combination of ZO with CMC did not improve the sorption properties, and all the ZO-CMC systems showed poor sorption properties compared with pure ZO for all contaminants. Figure 1*a*(iii,iv) reveal that in both the ZO-CTS and ZO-CMC composites an increase in the polymer content resulted in a lower adsorption capacity for both Pb(II) and Cd(II). During the synthesis of ZO-CTS and ZO-CMC, a slurry was formed when the polymer percentage exceeded 70% (w/w). This means there is an upper limit to the amount of the polymer that can be incorporated in the composite. Overall, the best sorption properties were shown at 30% w/w polymer and 70% w/w ZO in ZO-CTS. Therefore, it can be assumed that this ratio is the best ratio that can result in a homogeneous composite with optimum chelating properties.

Hence, all the characterization and further adsorption studies were carried out for this composite.

### 3.2. Morphological characterization

**Figure 2.**
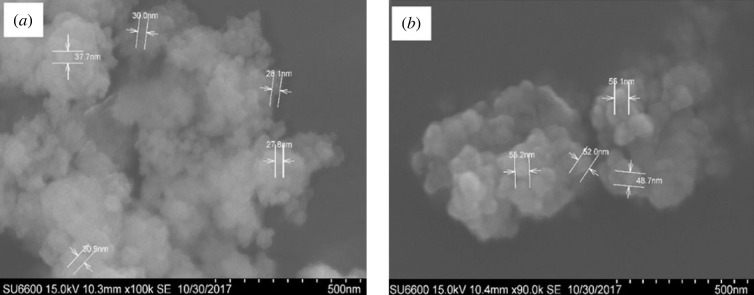
SEM images of (*a*) ZO and (*b*) ZO-CTS.

ZO and ZO-CTS were analysed using SEM (figure 2). It can be observed that the neat ZO nanoparticles lie in the size range of 20–40 nm. When CTS was added, the particle size increased to 48–55 nm. This increment in size was accompanied by a narrowing of the size distribution range. Similar observations have been made by Unsoy *et al.* [45] and Tran *et al.* [46].

### 3.3. Fourier transform infrared spectroscopy

**Figure 3.**
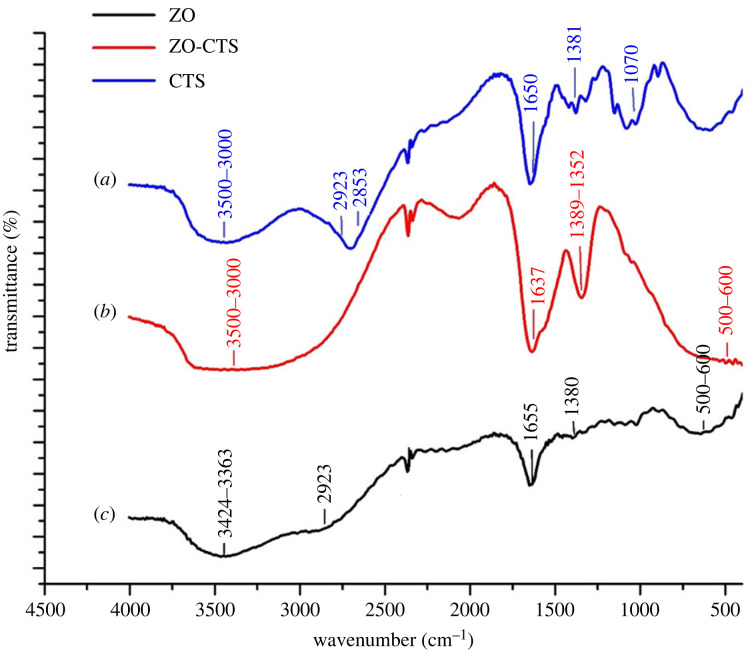
FT-IR spectra of (*a*) neat CTS, (*b*) ZO and (c) ZO-CTS.

FT-IR analysis was performed to identify the functional groups present in ZO and ZO-CTS. As shown in figure 3, the presence of several bands corresponding to both neat ZO and CTS in ZO-CTS confirms the formation of a composite material. The appearance of a broad band in the region of 3424–3363 cm^−1^ indicates the presence of –OH and –NH_2_ [47,48] and adsorbed water [22]. The peaks at 2923 and 2853 cm^−1^ can be assigned to CTS C-H asymmetric and symmetric stretching vibrational bands [44,47,49]. The presence of CTS is further confirmed from the –C-O stretching and C-H bends at around 1380 cm^−1^ [47]. Vibrations in the region 500–600 cm^−1^ correspond to Zr-O vibrations [48,50,51].

**Figure 4.**
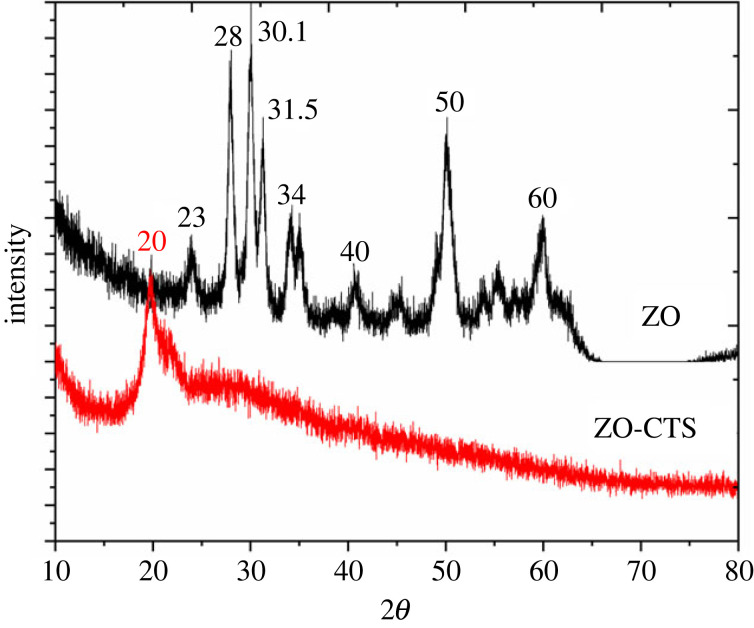
X-ray powder diffraction patterns of ZO and ZO-CTS.

Figure 4 shows the XRD patterns of ZO and ZO-CTS. The XRD pattern of ZO matches with the XRD pattern of crystalline monoclinic ZO [12,15,16]. The diffraction pattern of ZO was matched with ICDD database and it resulted in the diffraction pattern relevant to both monoclinic and tetragonal ZO corresponding to DB card number 01-078-3037 and 00-068-0200 in ICDD database. In electronic supplementary material, section S2, the relevant planes and the detailed information of the DB cards are given as indicated in the electronic supplementary material, figures S2.1 and S2.2. However, the diffraction pattern of ZO-CTS nanocomposite is not crystalline as ZO. This should be due to the amorphous nature of CTS. In addition to that, the diffraction pattern could not be matched with ICDD database as the resulting nanocomposite is a novel material. The XRD pattern of ZO-CTS shows only one broad peak at 2*θ* = 20°. It appears that the ZO in ZO-CTS is present in the amorphous form as observed in earlier reports [21,52]. This indicates that the particles depicted in the SEM images (figure 2*b*) comprise a CTS matrix with ZO nanoparticles dispersed through it.

**Figure 5.**
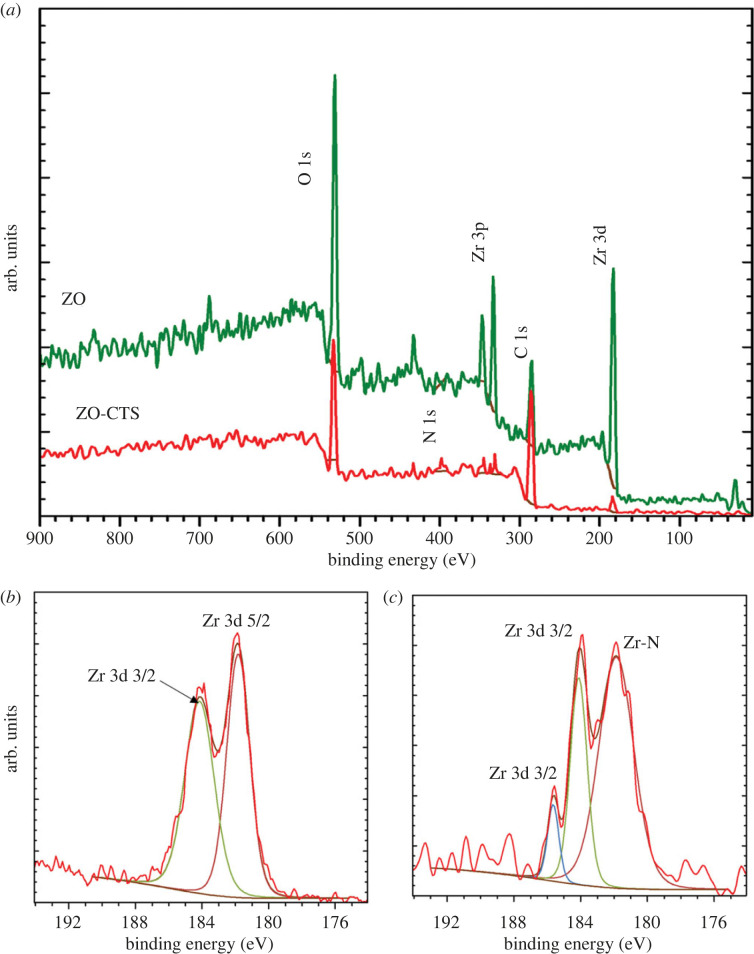
XPS spectra. (*a*) Overlaid ZO and ZO-CTS survey spectra, and high-resolution spectra of the Zr 3d region for (*b*) ZO and (*c*) ZO-CTS.

In order to estimate the surface elements and to obtain information about the chemical states of zirconium, XPS was performed. The survey spectra of ZO and ZO-CTS are overlaid in figure 5*a* and indicate the presence of the expected elements. The intensity of Zr peaks is decreased in ZO-CTS due to the presence of CTS diluting the Zr concentration in the sample. An unexpected C1s peak in ZO can be seen, which is attributed to an impurity.

Figure 5*b*,*c* shows the Zr-3d region. For ZO, (figure 5*b*) the characteristic peaks of Zr-3d_5/2_ and Zr-3d_3/2_ are seen at 182 and 184 eV, respectively. These peaks indicate the presence of only one oxidation state of Zr (IV) as expected based on the literature [53]. However, in the case of ZO-CTS, the Zr-3d peaks are split and new peaks are visible at 183.8 and 185.5 eV (figure 5*c*). This is thought to be due to the strong interaction of Zr with electronegative atoms (N and O) in the polymer [21,54].

### 3.4. Adsorption experiments on ZO and ZO-CTS

#### 3.4.1. Fluoride

The pH dependence of the adsorption of F^−^ ions by ZO and the ZO-CTS nanocomposite is shown in electronic supplementary material, figure S1. Adsorption of F^−^ by both ZO and ZO-CTS is highest at acidic pH. The adsorption decreased steadily with increasing pH and levelled off around pH = 5.5. At higher pH values, the adsorption capacity dropped dramatically. A pH of 5.5 thus can be considered as optimum. F^−^ absorption is efficient at acidic pH due to the low concentration of competing OH^−^ ions. The distribution of ZO through the CTS matrix in ZO-CTS and the interaction of Zr(IV) with hydroxyl and amine groups in CTS may play a synergistic role in improving the adsorption capacity of ZO-CTS for F^−^.

The adsorption of F^−^ ions as a function of time was measured by varying the contact time, over a range of 30 s to 2 h (electronic supplementary material, figure S2). Adsorption of fluoride on both ZO and ZO-CTS is characterized by initially rapid uptake, reaching equilibrium within a short time (approx. 10 min and approx. 4 min respectively). The adsorption of F^−^ by ZO-CTS (120 mg g^−1^) at equilibrium is four times as high as with ZO (59.8 mg g^−1^).

Fluoride adsorption by ZO and ZO-CTS was studied over the concentration range 40–350 ppm at pH = 5.5, and the results were fitted with four isotherm models (Langmuir, Freundlich, Temkin and DR isotherms). The resultant plots are given in the electronic supplementary material, figure S3, and the isotherm constants in table 1. The Freundlich isotherm provides the best fit for uptake by ZO, while the ZO-CTS data match best with Temkin adsorption. Both isotherms describe heterogeneous surfaces with the possibility of multi-layer type adsorption, which is sensible given the nature of the composite.

**Table 1.** Adsorption isotherm constants for F^−^, As(V), Pb(II) and Cd(II) adsorption of ZO-CTS and ZO.

adsorption parameter		F^−^		As(V)		Pb(II)		Cd(II)	
		ZO-CTS	ZO	ZO-CTS	ZO	ZO-CTS	ZO	ZO-CTS	ZO
experimental data	qe (mg g^−1^)	120.10	29.70	14.90	8.20	170.10	389.70	72.20	139.40
	s.d.	0.20	0.40	0.24	0.37	0.80	0.80	0.60	0.90
Langmuir isotherm	*Q*_L_ (mg g^−1^)	138.89	39.22	18.08	15.31	243.90	434.78	99.01	270.27
	*K*_L_ (min^−1^)	41.29	94.90	3.06	11.21	677.20	103.39	45.97	502.84
	SSE	0.02	0.06	0.03	0.22	0.09	0.01	0.07	0.23
	*R*^2^	1.00	0.98	0.97	0.99	0.91	0.99	0.96	0.96
Freundlich isotherm	*Q*_F_ (mg g^−1^)	85.74	78.80	15.51	5.61	173.81	412.07	72.20	141.11
	*K*_f_ (g mg^−1^ min^−1^)	0.32	0.08	4.34	1.22	3.71	23.07	2.39	4.23
	*n*	0.98	1.93	1.89	1.77	1.90	2.24	1.76	1.38
	SSE	0.161	0.388	0.002	0.213	0.000	0.003	0.000	0.000
	*R*^2^	0.99	0.99	0.99	0.93	0.96	0.99	0.98	1.00
Temkin isotherm	*K*_t_ (l g^−1^)	0.26	1.46	1.48	1.22	0.02	0.13	0.06	0.12
	*B*_T_ (J mol^−1^)	29.15	4.16	3.41	3.06	49.61	82.57	21.39	46.23
	*q*_T_ (mg g^−1^)	141.64	21.16	10.56	9.44	163.10	365.72	66.38	126.01
	SSE	0.023	0.163	0.169	0.017	0.002	0.004	0.008	0.011
	*R*^2^	1.00	0.97	0.95	0.98	0.91	0.98	1.00	0.94
Dubnin–Rarushkevish isotherm	*K*_DR_ (mol^2^ J^−2^)	3 × 10^−5^	2 × 10^−4^	1 × 10^−7^	1 × 10^−8^	5 × 10^−5^	1.8 × 10^−3^	0.00	0.00
	Qm (mg g^−1^)	92.01	40.40	10.15	7.47	120.86	254.42	55.26	97.82
	*E* (kJ mol^−1^)	0.09	0.35	1.41	0.00	0.06	0.00	0.06	0.01
	*R*^2^	0.78	0.77	0.80	0.85	0.58	0.72	0.86	0.81

**Figure 6.**
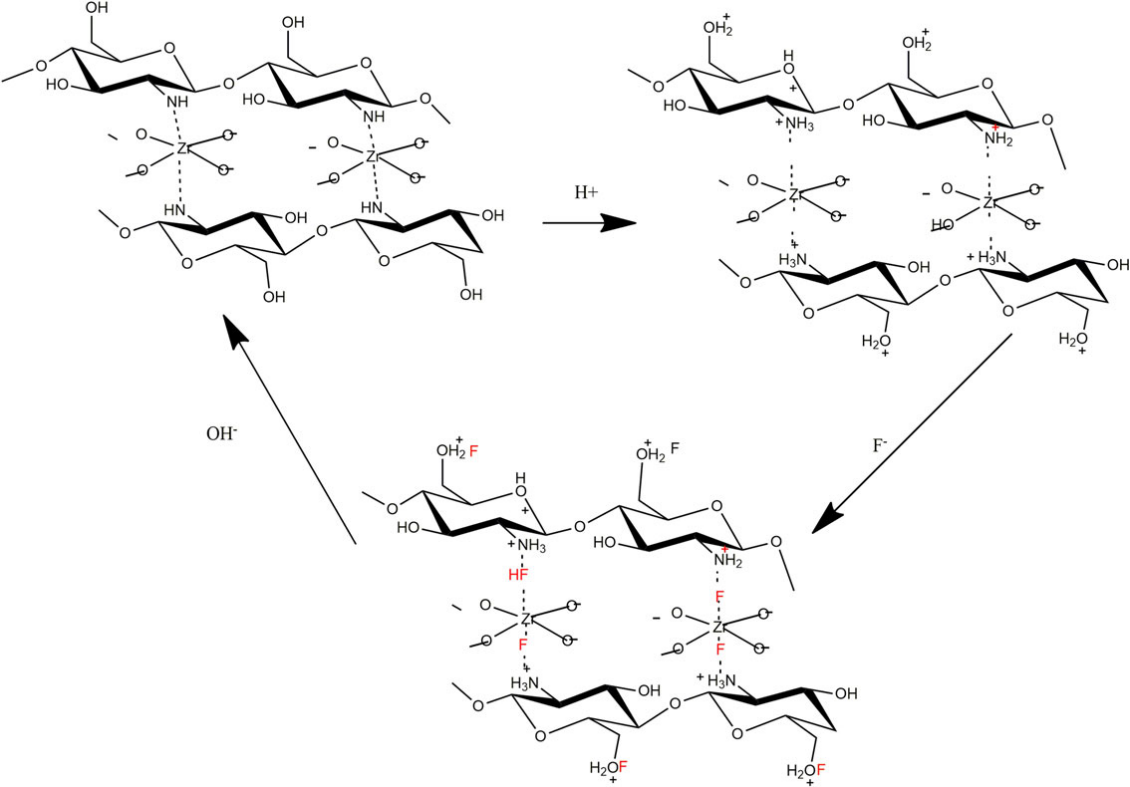
Suggested mechanism for fluoride adsorption by ZO-CTS.

Figure 6 shows the suggested mechanism for fluoride adsorption by ZO-CTS. Ion exchange sites are proposed to arise at acidic pH when both -NH_2_ and -OH groups in CTS are protonated, facilitating the adsorption of fluoride ions onto the surface.

#### 3.4.2. Arsenate

**Figure 7.**
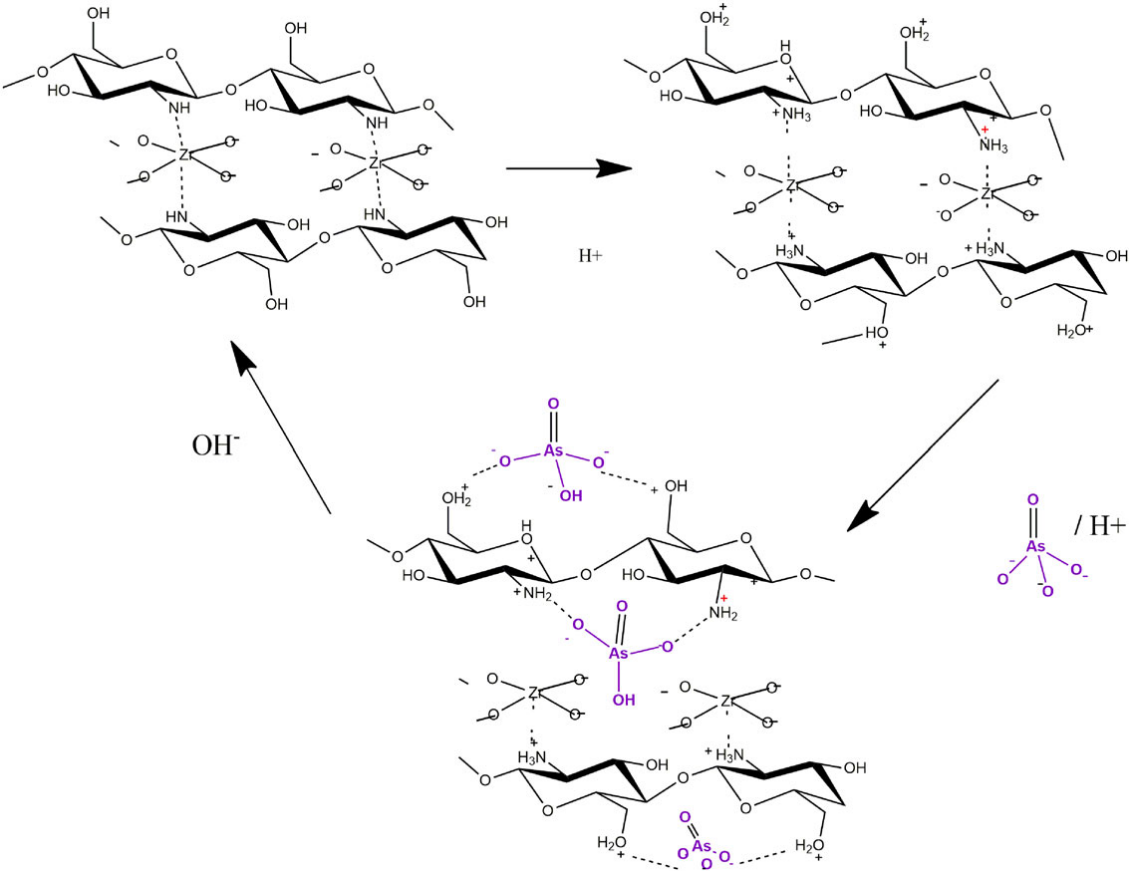
Suggested mechanism for As(V) adsorption on ZO-CTS.

The effect of solution pH on the adsorption of As(V) by ZO and ZO-CTS was tested over the pH range of 1 to 11. The results are presented in electronic supplementary material, figure S4. At all the pH values, ZO-CTS shows improved adsorption properties over ZO. With both materials, improved adsorption properties can be observed at acidic pH, and the highest adsorption capacity for ZO is observed at around pH = 4.8. ZO-CTS shows a considerably higher adsorption capacity at around pH 5.5 with only a small further capacity increase of around 1% at pH values below that. In basic media, adsorption of As(V) decreases due to competition from OH^−^ ions. The surface of the adsorbent can be expected to be negatively charged due to the adsorption of OH^−^ ions at high pH. The time dependence of As(V) uptake was measured within the time range of 30 s to 2 h (see electronic supplementary material, figure S5). It can be observed that the adsorption of As(V) is greater on ZO-CTS than on ZO. Adsorption of As(V) on both ZO and ZO-CTS matrices is characterized by fast kinetics (10 and 5 min) Fits of isotherm models to the As(V) uptake data are shown in the electronic supplementary material, figure S6, with the calculated parameters presented in table 1. The maximum As(V) adsorption capacity at 14.87 mg g^−1^ was observed for ZO-CTS and for ZO it was 9.9 mg g^−1^. As(V) adsorption on ZO-CTS is best fitted with the Freundlich isotherm. By contrast, uptake by ZO showed agreement with the Langmuir model. In general, As(V) adsorption on both ZO and ZO-CTS showed relatively good agreement with all three adsorption models (Langmuir, Freundlich and DR). On this basis, a suggested mechanism for As(V) adsorption on ZO-CTS is illustrated in figure 7. The possibility of negative arsenate ions interacting with protonated -NH_2_ (${\mathrm{NH}}_{3}^{+}$) and OH ($-{\mathrm{OH}}_{2}^{+}$) groups of CTS (in an acidic medium) is thought to be key to uptake.

#### 3.4.3. Pb(II) and Cd(II)

The adsorption of Cd(II) and Pb(II) is depicted in electronic supplementary material, figures S7 and S8, respectively. In contrast with As(V) and fluoride, adsorption of Cd(II) and Pb(II) increased with a rising solution pH. For both Pb(II) and Cd(II), uptake by ZO is higher than with ZO-CTS across the pH range studied. It can be suggested that when ZO is incorporated into CTS, the number of active sites for Cd(II) and Pb(II) adsorption is decreased due to the interactions of ZO with CTS. It is known that the surface of ZO is negatively charged and associated with hydroxyl groups [55]. In the composite, the Zr sites are likely to be covered by the polymer, hindering access to them by Pb(II) and Cd(II).

Adsorption of Pb(II) and Cd(II) was investigated using aqueous solutions containing 2197 ppm and 547 ppm, respectively (electronic supplementary material, figures S9 and S10). While ZO displayed a higher adsorption capacity, ZO-CTS showed more rapid uptake kinetics compared with ZO. In order to identify the type of adsorption mechanisms, both ZO and ZO-CTS were subjected for batch adsorption studies and the adsorption isotherms plotted according to the Langmuir, Freundlich, Temkin and DR isotherm models (electronic supplementary material, figures S12 and S13 and table 1).

It was found that the adsorption capacities of ZO and ZO-CTS for Pb(II) were 389.7 and 170 mg g^−1^, respectively, while for Cd(II) the equivalent values are 434.7 and 173.8 mg g^−1^. The Freundlich model fits the uptake of both ions by both composites. This indicates the presence of a heterogeneous surface and multi-layer adsorption mechanism.

In this work, improved adsorption by the CTS incorporation into ZO was observed only for F^−^ and As(V). Further studies were thus carried out to analyse the post-adsorbed samples and also to investigate their applicability in real applications.

### 3.5. Energy-dispersive X-ray spectroscopy analysis of F^−^ and As(V) adsorbed ZO-CTS and ZO

EDEX spectra of fresh ZO-CTS and ZO and after adsorption of F^−^ and As(V) are shown in the electronic supplementary material, figure S13. The spectra indicate the successful synthesis of ZO and ZO-CTS by the elemental composition. The EDEX of ZO-CTS and ZO after adsorption of F^−^ and As(V) show the former to contain a higher percentage of F and As than the latter, indicating its improved sorption properties.

### 3.6. The effect of other ions

**Figure 8.**
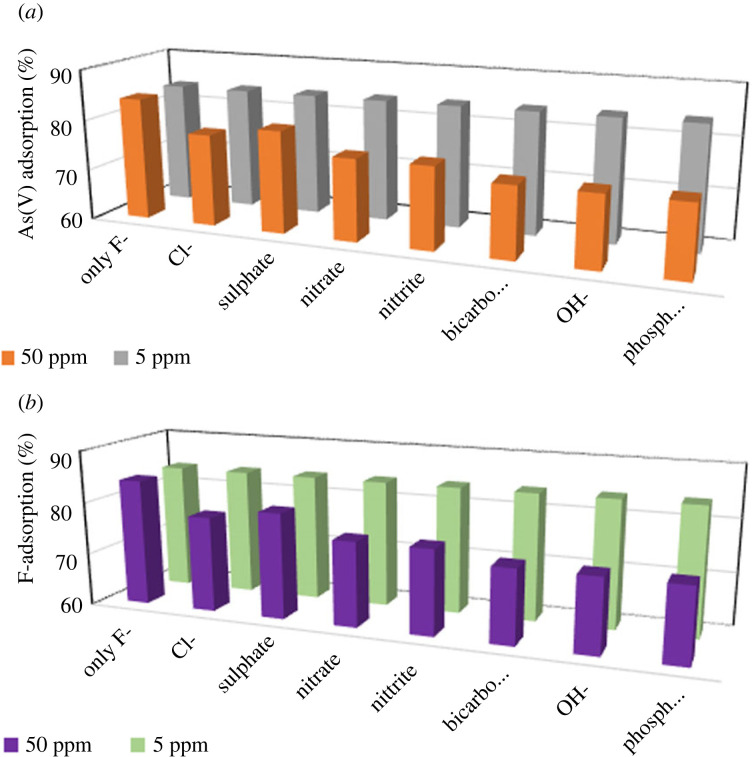
(*a*) As(V) and (*b*) fluoride adsorption capacity in the presence of other ions at 5 and 50 ppm.

The effect of other anions (chloride, sulphate, nitrate, nitrite, bicarbonate, hydroxyl and phosphate) on the adsorption of F^−^ and As(V) onto ZO-CTS were investigated and the results are graphically illustrated in figure 8. It was noticed when the concentration of those ions is adjusted to 5 ppm, there is almost no reduction in the adsorption percentages. However, when those ions are present at higher concentrations some inhibition in the adsorption of both F^−^ and As(V) onto ZO-CTS can be seen due to the competitiveness with these added anions. In particular, it can be clearly seen that in the presence of bicarbonate and hydroxyl ions, the adsorption capacity is lower than with other anions This can be easily ascribed to the increment of the pH of the system whereby losing its available positive charges on CTS sites for F^−^ and As(V) to bind with.

### 3.7. Reusability studies

Experiments were conducted to test the reusability of the ZO-CTS for F^−^ and As(V) adsorption over five cycles and the resulted plot is shown in the electronic supplementary material, figure S14. These revealed that ZO-CTS could be recovered and re-used, with only a 2–3% loss of capacity after five uptake cycles.

### 3.8. Gravity filtration studies

**Figure 9.**
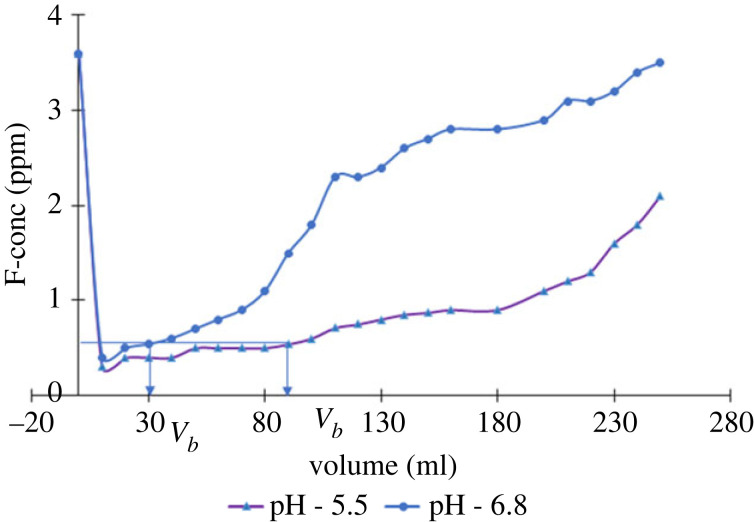
Breakthrough curve for gravity filtration studies of fluoride adsorption onto ZO-CTS.

**Figure 10.**
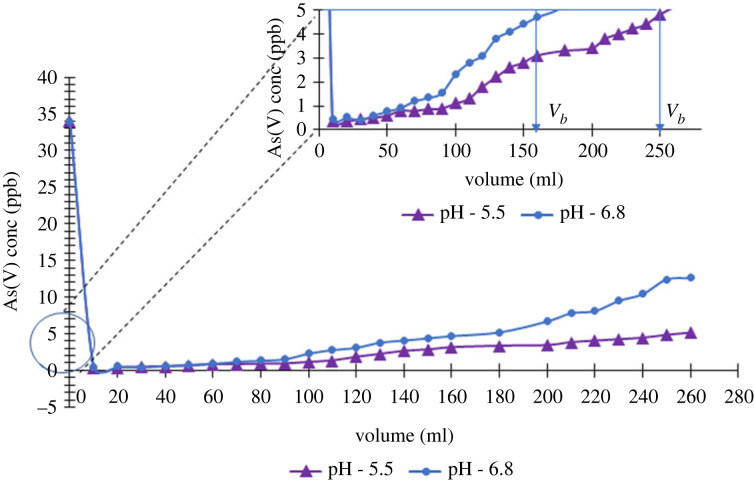
Breakthrough curve for gravity filtration studies of As(V) adsorption onto ZO-CTS.

Gravity filtration studies were conducted for a mixture of 3.6 ppm F^−^ and 34 ppb of As(V) ion solution, prepared by spiking actual groundwater samples. These concentrations were adjusted to match with the reported levels in CKDu prominent areas in Sri Lanka [5,6]. The inherent pH of the pollutant solutions was pH 6.8 and the gravity filtration studies were conducted at this pH without adjusting it. In order to compare the results, gravity filtration studies were also conducted at pH 5.5 as the optimum adsorption for both contaminants was found to be at this pH. The safe limits of F^−^ and As(V) were considered to be 0.5 ppm and 3 ppb based on the literature [39–41]. Figure 9 shows a plot of fluoride concentration versus the volume of solution passed through the gravity column at pH = 5.5 and pH = 6.8. The volume where the F^−^ concentration exceeds the safe limit is defined as the breakthrough volume (*V_b_*). At pH = 5.5, a *V_b_* of 90 ml was obtained for 0.05 g of adsorbent (1800 ml g^−1^). At pH = 6.8 the breakthrough volume was 30 ml (600 ml g^−1^). The breakthrough curve resulting for the gravity filtration studies for As(V) is given in figure 10. In acidic conditions (pH 5.5) *V_b_* is 250 ml for 0.05 g (12 500 ml g^−1^). However, at pH 6.8, the column becomes saturated faster (*V_b_* = 160 ml), indicating the reduced number of adsorption sites available, and the breakthrough capacity was 8000 ml g^−1^.

We, then compared our findings with relevant work published already and found that several studies have been conducted to investigate the adsorption properties of ZO-based materials including ZO-incorporated CTS. The summary of all these findings with our data is given in table 2. In comparison with the reported work, ZO-CTS synthesis in this work can be identified as a quite promising material in removing both F^−^ and As(V) from water, as it resulted a higher adsorption capacity with a shorter contact time compared with published reports. In addition, none of the studies have conducted gravity filtration, considering the actual concentration levels in the groundwater. Results obtained for the gravity filtration studies conducted in this work indicated the potential of ZO-CTS to be used in real water purification applications.

**Table 2.** Comparison of the adsorption properties of ZO-CTS for F^−^ and As(V) in this work with the reported work.

F^−^ adsorbent	adsorption capacity mg g^−1^	time (min)	initial concentration (ppm)	isotherm model	pH	gravity filtration studies	ref
F^−^							
Zr-doped CTS beads	3.27	24 × 60	5.65	Freundlich	6–6.9	—	[36]
Zr carbon hybrid sorbents	17.7	50	40	Langmuir	7	—	[51]
Zr-impregnated collagen	0.95		0.43	Langmuir		—	[56]
granular zirconium-iron oxide prepared using the extrusion method Zr/Fe molar ratio of 2/3	9.80	180	10.0	Freundlich	7	—	[57]
Zr-impregnated coconut fibre	40.016	360	—	Langmuir	4	—	[57]
Zr-impregnated coconut shell	6.41	360	10	Langmuir	4	—	[58]
ZO zeolite nanocomposites	0.36	480	5	Freundlich	6	—	[59]
zirconia-incorporated chitosan (ZO-CTS)	120	4	350	Temkin	5.5	initial concentration is 5 ppm; at pH 5.5, 1800 ml g^−1^; At pH 6.8, 600 ml g^−1^	this work
As (V)							
ZO nanoparticles	3.6	10	0.01	Langmuir	7	—	[22]
graphene oxide-ZO	84.8	15	80	Langmuir	7	—	[60]
	2	480	10	Langmuir	7	—	[61]
zirconia-incorporated chitosan (ZO-CTS)	14.87	5	50 ppm	Langmuir	5.5	initial concentration is 35 ppb; at pH 5.5, 12 500 ml g^−1^; at pH 6.8, 8000 ml g^−1^	this work

## 4. Conclusion

Synthesis of nano-ZO and composites of nano-ZO with CTS and CMC was achieved using a facile microwave method. The combination of ZO and CTS had enhanced the adsorption capacity for F^−^ and As(V). However, no such enhancement was observed for Pb(II) and Cd(II). Combination of ZO with CMC showed no value in using it as a sorbent, as it had very low adsorption capacities which are even lower than neat ZO. The ZO-CTS formulation with a 70/30 w/w composition was selected as the optimal formulation and explored in detail. The material was found to comprise nanoscale particles with ZO present in the amorphous form. Interactions between Zr and the amide groups of CTS were inferred from XPS data. In detailed pollutant uptake studies, it was observed that acidic conditions favour the adsorption of F^−^ and As(V). Gravity filtration studies showed that the ZO-CTS system has the potential to be used as an adsorbent to remove F^−^ and As(V) from water.

## Data Availability

The datasets supporting this article have been uploaded as part of the electronic supplementary material [62]. In addition the datasets can also be found at https://doi.org/10.5061/dryad.b8gtht7cv [63].
